# MS-YOLOv11: A Wavelet-Enhanced Multi-Scale Network for Small Object Detection in Remote Sensing Images

**DOI:** 10.3390/s25196008

**Published:** 2025-09-29

**Authors:** Haitao Liu, Xiuqian Li, Lifen Wang, Yunxiang Zhang, Zitao Wang, Qiuyi Lu

**Affiliations:** Department of Astronautics, Space Engineering University, Beijing 101416, China; liuhaitao@hgd.edu.cn (H.L.);

**Keywords:** multi-scale features, wavelet transform, remote sensing image, YOLOv11, small objects detection

## Abstract

In remote sensing imagery, objects smaller than 32×32 pixels suffer from three persistent challenges that existing detectors inadequately resolve: (1) their weak signal is easily submerged in background clutter, causing high miss rates; (2) the scarcity of valid pixels yields few geometric or textural cues, hindering discriminative feature extraction; and (3) successive down-sampling irreversibly discards high-frequency details, while multi-scale pyramids still fail to compensate. To counteract these issues, we propose MS-YOLOv11, an enhanced YOLOv11 variant that integrates “frequency-domain detail preservation, lightweight receptive-field expansion, and adaptive cross-scale fusion.” Specifically, a 2D Haar wavelet first decomposes the image into multiple frequency sub-bands to explicitly isolate and retain high-frequency edges and textures while suppressing noise. Each sub-band is then processed independently by small-kernel depthwise convolutions that enlarge the receptive field without over-smoothing. Finally, the Mix Structure Block (MSB) employs the MSPLCK module to perform densely sampled multi-scale atrous convolutions for rich context of diminutive objects, followed by the EPA module that adaptively fuses and re-weights features via residual connections to suppress background interference. Extensive experiments on DOTA and DIOR demonstrate that MS-YOLOv11 surpasses the baseline in mAP@50, mAP@95, parameter efficiency, and inference speed, validating its targeted efficacy for small-object detection.

## 1. Introduction

With the rapid advancement of remote sensing technology, its applications in environmental monitoring [1], urban planning [2], and disaster assessment [3] have become increasingly widespread. In recent years, the application of deep learning techniques has significantly enhanced the feature extraction capabilities of convolutional neural networks [4]. Currently, mainstream object detection algorithms are divided into two-stage and single-stage detection methods [5]. Among them, the YOLO series [6] of single-stage detectors exhibits great potential in improving recognition accuracy and has demonstrated strong practicality and broad application potential across various computer vision tasks. Moreover, the real-time processing capability and computational efficiency of YOLO series algorithms provide significant advantages for deployment in aerospace systems [7]. With continuous iteration and optimization of the YOLO architecture, it can better adapt to general object detection tasks.

However, objects in remote sensing images are often extremely small, and their distinguishable features are limited due to high-altitude acquisition, which restricts effective feature extraction. Secondly, cluttered backgrounds combined with environmental factors such as weather variations and illumination differences exacerbate foreground–background confusion and reduce detection accuracy. Furthermore, the lack of texture and shape cues can lead to confusion among visually similar small objects (e.g., vehicles vs. oil tanks, ships vs. floating bridges). Meanwhile, weak target signals are easily overshadowed by background clutter, resulting in a significantly higher missed detection rate compared to medium and large objects [8]. Therefore, the accurate detection of small objects [9] in remote sensing images using YOLO series algorithms remains a major challenge. In summary, the core challenges of small object detection in such scenarios can be categorized into the following three aspects:

1. Weak target signals: Objects are easily obscured in wide fields of view and strong clutter, leading to a high missed detection rate.

2. Insufficient pixel information: Extreme scarcity of geometric contours and texture details makes it difficult for traditional convolution operations to capture discriminative representations.

3. Loss of high-frequency information: Continuous downsampling operations irreversibly eliminate edge and texture details, while existing pyramid structures still struggle to compensate for fine-grained structural information. To address these challenges, this paper proposes MS-YOLO (Multi-Scale YOLO), designed for remote sensing scenarios. Based on the YOLOv11 baseline, the algorithm introduces three innovative strategies: preservation of frequency domain detail, lightweight receptive field expansion, and adaptive cross-scale fusion. The specific contributions are as follows:

1. Frequency-domain detail preservation: A 2D Haar wavelet transform is embedded at the input stage to explicitly decompose the image into a low-frequency approximation component and three high-frequency detail sub-bands. This process suppresses background noise while highlighting subtle edges and textures that are prone to being obscured.
2. Lightweight receptive field expansion: Small-kernel depthwise separable convolutions are independently applied to each sub-band. This approach exponentially expands the receptive field without introducing redundant parameters, preserving global context while avoiding excessive smoothing of small objects.
3. Adaptive cross-scale fusion: A Mix Structure Block (MSB) is designed, which includes the following:
  - An MSPLCK module that employs parallel dilated convolutions (7×7→13×13→19×19) for dense sampling, capturing multi-scale contextual information of extremely small targets;
  - An EPA module that utilizes dual channel-space attention mechanisms with residual connections to adaptively weight features, dynamically suppressing background interference and enhancing target features.

## 2. Related Work

### 2.1. Object Detection Methods

The research landscape of object detection has demonstrated a distinct evolutionary trend from traditional methods to deep learning techniques. Early conventional approaches primarily relied on combinations of handcrafted feature extractors and classifiers. Template matching methods such as the Viola–Jones detector (2001) [10] and HOG+SVM (2005) [11] achieved object localization through sliding window mechanisms combined with Haar features or histogram of oriented gradients, but suffered from high computational redundancy and poor real-time performance.

With the rise of deep learning, object detection technology has undergone fundamental transformation. The end-to-end learning paradigm based on convolutional neural networks (CNNs) has significantly improved detection accuracy and efficiency. Modern deep learning approaches primarily follow three technical pathways: two-stage detection [12], single-stage detection [13], and anchor-free detection. Two-stage methods first generate region proposals followed by classification and regression, with representative works including the R-CNN series (e.g., Fast R-CNN, Faster R-CNN) [14], and subsequent developments such as FPN (enhancing multi-scale feature fusion through feature pyramid networks) [15,16,17] and Mask R-CNN [18] (extended to instance segmentation tasks). These methods achieve high detection accuracy suitable for scenarios requiring strict precision, albeit with higher computational complexity. Single-stage methods like the YOLO series and SSD [19] directly predict object categories and locations on feature maps, trading some accuracy for speed advantages, making them more suitable for real-time applications. The YOLO series has undergone multiple iterations from YOLOv1 to the latest YOLOv12, incorporating technologies such as multi-scale prediction [20], CSPNet backbone networks [21], Transformer attention mechanisms [22], and neural architecture search (NAS) [23], continuously balancing precision and efficiency. Anchor-free methods [24] (e.g., CenterNet, CornerNet, and DETR) abandon preset anchor boxes, directly predicting key points or utilizing self-attention mechanisms for end-to-end detection, reducing hyperparameter dependency but facing challenges such as slow training convergence and high computational demands.

YOLOv11 demonstrates an optimal balance among accuracy, speed, and extensibility, making it particularly suitable for the challenging task of small object detection in remote sensing images. In contrast, although YOLOv10 [25] performs excellently in general object detection tasks—such as safety helmet monitoring on construction sites as referenced in related studies—these tasks typically involve larger objects and relatively simple backgrounds. Consequently, its architectural design and focus are not fully aligned with our requirements for multi-scale feature extraction, high-frequency detail preservation, and lightweight extensibility. Therefore, selecting YOLOv11 as the baseline and implementing targeted enhancements is both justified and effective.

### 2.2. Deep Learning Algorithms for Small Object Detection

The detection of small objects in remote sensing imagery represents a critical challenge in the field of computer vision. In recent years, researchers worldwide have conducted extensive studies across multiple directions, including feature enhancement and attention mechanisms, multi-scale feature fusion, model lightweighting and efficient detection, as well as Transformer-based novel approaches, significantly advancing the development of this field.

In terms of feature enhancement, researchers have improved the representational capacity for small objects by introducing attention mechanisms and feature reconstruction techniques. For instance, Huang et al., building upon Faster R-CNN, employed image upsampling and gradient ascent methods to reconstruct features while preserving detailed information. Wu et al. integrated spatial-to-depth transformations with attention mechanisms, enhancing both accuracy and the ability to distinguish densely distributed objects. However, such methods often incur substantial computational overhead, making it difficult to meet real-time detection requirements.

Multi-scale feature [26] fusion represents a crucial technical pathway for addressing scale variations. Shan et al. combined Region Proposal Networks (RPNs) with multi-angle RoI extraction [27], effectively leveraging multi-level features. Lv et al. proposed the RT-DETR model, which enhances multi-scale processing capabilities through cross-scale fusion and IoU-aware query selection mechanisms [28]. Liu et al. further designed SO-RTDETR [29], incorporating attention mechanisms to balance detection performance across objects of different scales. Although these methods achieve excellent accuracy, their complex structures and high training costs demand substantial computational resources.

Additionally, a series of improved algorithms based on the YOLO architecture have accelerated the advancement of small object detection. For example, LGFF-YOLO [30] enhances multi-scale feature representation through a Global Information Fusion Module (GIFM) and a Four-Leaf Clover Fusion Module (FLCM). FFCA-YOLO [31] introduces a Feature Enhancement Module (FEM) to expand the receptive field, while SF-YOLO [32] optimizes foreground–background discrimination via a Spatial Information Perception (SIP) module. Meanwhile, novel paradigms such as Mutual Learning (MA) [33] further improve detection performance through branch collaboration and feature alignment. Examples include MADet [34], which employs hybrid regression strategies to handle diverse objects, and DICN [35], which facilitates cross-domain knowledge transfer through network consistency [36].

In summary, CNNs—particularly YOLOv11—provide a strong baseline for small object detection in remote sensing, offering a balanced combination of speed, accuracy, and practical deployability. Their inductive bias toward local feature extraction aligns well with the core challenges of this task. While transformers [37] hold theoretical advantages in global modeling, their computational cost may be prohibitive for many real-time remote sensing applications. Therefore, MS-YOLOv11 adopts a hybrid approach: it enhances local detail extraction via wavelet transform and compensates for global context limitations through efficient attention and multi-scale mechanisms. This results in simultaneous improvements in both accuracy and speed, better meeting the practical demands of small object detection in remote sensing imagery.

## 3. Proposed Model

### 3.1. Overview of MS-YOLO Model

The original YOLOv11 architecture employs four C3K2 modules in its backbone network for feature extraction, which may induce feature degradation. To mitigate this limitation, we integrate a wavelet transform convolutional neural network (WTCNN) module into the backbone. This innovative module performs multi-scale target decomposition through wavelet transformation, enabling effective capture of target features across different scales. The proposed enhancement not only improves small object detection accuracy but also enhances multi-scale target detection performance in complex scenarios.

**Figure 1:**
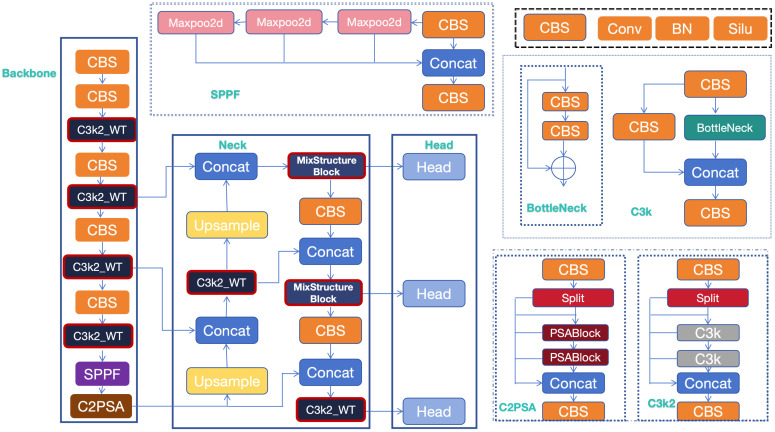
Architectural overview of MS-YOLOv11 network.

Within the neck network, the Mix Structure Block module plays a pivotal role in multi-scale feature fusion. By combining feature fusion techniques with residual connections, this module optimizes feature representation while capturing multi-scale characteristics. This design significantly improves feature robustness and discriminative power, enabling superior performance in detection tasks involving complex backgrounds and variable illumination conditions. The comprehensive network architecture is illustrated in Figure 1.

### 3.2. Algorithm Principle of Wavelet Transform

While the Fourier transform decomposes signals into global sinusoidal components, this approach presents limitations for analyzing image features that exhibit strong spatial locality, such as edges and texture patterns. Consider an image containing fine details in its upper-left quadrant versus smooth regions in the lower-right quadrant: these distinct areas demand independent spatial analysis, yet the Fourier transform inherently lacks spatial localization capabilities. This limitation becomes particularly evident when processing non-stationary image signals—for instance, abrupt frequency transitions at object boundaries—where the Fourier transform can only yield an aggregated frequency spectrum without indicating the spatial distribution of high-frequency edge components [38]. In remote sensing target detection applications, spatial localization proves especially critical. Essential image features like object boundaries manifest as localized high-frequency components. The wavelet transform addresses this fundamental limitation by directly encoding spatial position information (through image coordinates) in its high-frequency subbands (LH, HL, and HH). This stands in contrast to Fourier analysis, which merely indicates the global presence of high-frequency content without spatial reference. Moreover, wavelet decomposition naturally generates a pyramid-structured representation (exemplified by the Laplacian pyramid), facilitating hierarchical, coarse-to-fine feature extraction. This multi-resolution property makes wavelet analysis particularly suitable for computer vision tasks including target detection and image fusion. The mathematical framework of the two-dimensional Haar wavelet transform is formally presented in Algorithm 1.
**Algorithm 1** 2D Haar Wavelet Transform for Image Decomposition**Require:** 
Input image $A\in R^{C\times H\times W}$ with *C* channels, *H* and *W* even**Ensure:** 
Decomposed representation $H_{\mathrm{HWT}}\in R^{4C\times H/2\times W/2}$1:Initialize low-pass filter $f_{L}=\frac{1}{\sqrt{2}}\left[1,1\right]$2:Initialize high-pass filter $f_{H}=\frac{1}{\sqrt{2}}\left[1,-1\right]$3:**for** c=1 to *C* **do**                   ▹ Process each channel4:    Initialize temporary tensors: $spec_{L},spec_{H}\in R^{H/2\times W}$5:    **for** i=0 to H/2−1 **do**6:        **for** j=0 to W−1 **do**7:           $spec_{L}\left[i\right]\left[j\right]\leftarrow f_{L}\left[0\right]\;\cdot \;A\left[c\right]\left[2i\right]\left[j\right]+f_{L}\left[1\right]\;\cdot \;A\left[c\right]\left[2i+1\right]\left[j\right]$8:           $spec_{H}\left[i\right]\left[j\right]\leftarrow f_{H}\left[0\right]\;\cdot \;A\left[c\right]\left[2i\right]\left[j\right]+f_{H}\left[1\right]\;\cdot \;A\left[c\right]\left[2i+1\right]\left[j\right]$9:        **end for**10:    **end for**11:    Initialize output subbands: $LL,LH,HL,HH\in R^{H/2\times W/2}$12:    **for** i=0 to H/2−1 **do**13:        **for** j=0 to W/2−1 **do**14:           $LL\left[i\right]\left[j\right]\leftarrow f_{L}\left[0\right]\;\cdot \;spec_{L}\left[i\right]\left[2j\right]+f_{L}\left[1\right]\;\cdot \;spec_{L}\left[i\right]\left[2j+1\right]$15:           $LH\left[i\right]\left[j\right]\leftarrow f_{H}\left[0\right]\;\cdot \;spec_{L}\left[i\right]\left[2j\right]+f_{H}\left[1\right]\;\cdot \;spec_{L}\left[i\right]\left[2j+1\right]$16:           $HL\left[i\right]\left[j\right]\leftarrow f_{L}\left[0\right]\;\cdot \;spec_{H}\left[i\right]\left[2j\right]+f_{L}\left[1\right]\;\cdot \;spec_{H}\left[i\right]\left[2j+1\right]$17:           $HH\left[i\right]\left[j\right]\leftarrow f_{H}\left[0\right]\;\cdot \;spec_{H}\left[i\right]\left[2j\right]+f_{H}\left[1\right]\;\cdot \;spec_{H}\left[i\right]\left[2j+1\right]$18:        **end for**19:    **end for**20:    Stack subbands: $H_{\mathrm{HWT}}\left[4c:4c+3\right]\leftarrow \left[LL,LH,HL,HH\right]$21:**end for**22:**return** $H_{\mathrm{HWT}}$

This work utilizes the Haar wavelet transform (WT) primarily for its computational efficiency and implementation advantages. We emphasize that our methodology is not fundamentally constrained to this specific wavelet basis, as other wavelet families could alternatively be implemented, though with potentially greater computational demands.

For a given image X, the single-level one-dimensional Haar WT (applied along either width or height dimensions) is implemented through: (1) depthwise convolution using the analysis kernels $\frac{1}{\sqrt{2}}\left[1,1\right]$ and $\frac{1}{\sqrt{2}}\left[1,-1\right]$, followed by (2) standard downsampling with stride 2. The two-dimensional extension combines these operations along both spatial dimensions, effectively realized as a depthwise convolution with stride 2 employing four distinct filter sets:

(1) $$\begin{array}{cc} f_{LL}=\frac{1}{2}\left[\begin{array}{cc} 1 & 1\\ 1 & 1 \end{array}\right], & f_{LH}=\frac{1}{2}\left[\begin{array}{cc} 1 & -1\\ 1 & -1 \end{array}\right],\\ f_{HL}=\frac{1}{2}\left[\begin{array}{cc} 1 & 1\\ -1 & -1 \end{array}\right], & f_{HH}=\frac{1}{2}\left[\begin{array}{cc} 1 & -1\\ -1 & 1 \end{array}\right] \end{array}$$

Here, $f_{LL}$ operates as a low-pass filter, while $f_{LH}$, $f_{HL}$ and $f_{HH}$ constitute complementary high-pass filters. The convolutional operation for each input channel yields the following:

(2) $$\left[X_{LL},X_{LH},X_{HL},X_{HH}\right]=\mathrm{Conv}\left(\left[f_{LL},f_{LH},f_{HL},f_{HH}\right],X\right),$$

producing four output channels with each channel’s spatial resolution reduced by half relative to X. The components $X_{LL}$, $X_{LH}$, $X_{HL}$, and $X_{HH}$ respectively represent the low-frequency approximation and horizontal, vertical, and diagonal high-frequency details. The orthogonal nature of the basis filters in Equation (1) permits exact reconstruction via transposed convolution:

(3) $$X=\mathrm{ConvTranspose}\left(\left[f_{LL},f_{LH},f_{HL},f_{HH}\right],\left[X_{LL},X_{LH},X_{HL},X_{HH}\right]\right)$$

Cascaded wavelet decomposition is achieved through recursive application to the low-frequency components:

(4) $$X_{LL}^{\left(i\right)},X_{LH}^{\left(i\right)},X_{HL}^{\left(i\right)},X_{HH}^{\left(i\right)}=\mathrm{WT}\left(X_{LL}^{\left(i-1\right)}\right)$$

where superscript (i) denotes decomposition level. This hierarchical process progressively enhances frequency resolution while reducing spatial resolution for low-frequency components.

Notably, convolutional operations in the wavelet domain enable expanded receptive fields. For instance, applying a 3×3 convolution to the second-level low-frequency component $X_{LL}^{\left(2\right)}$ effectively establishes a nine-parameter kernel operating on a 12×12 receptive field in the original input X for low-frequency signal processing.

### 3.3. Wavelet Transform Convolutional Neural Network

**Figure 2:**
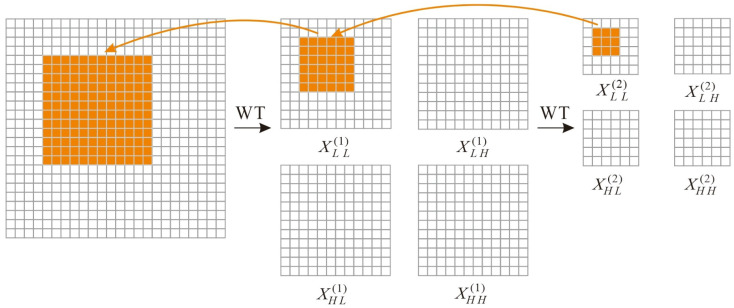
Operational principle of wavelet transform convolutional neural network.

The quadratic growth in parameters associated with increasing convolutional kernel sizes presents a significant challenge. Our solution involves a three-stage process: (1) wavelet-based filtering and downsampling of input low/high-frequency components, (2) small-kernel depthwise convolution on frequency-specific feature maps, and (3) output reconstruction via inverse wavelet transform. Mathematically, this operation is expressed as:

(5) $$Y=\mathrm{IWT}\left(\mathrm{Conv}\left(W,\mathrm{WT}(X)\right)\right)$$

where *X* denotes the input tensor and *W* represents the weight tensor of the k × k depthwise convolution kernel, with input channels quadruple those of X. This approach not only isolates frequency-specific convolutions but also enables small kernels to operate on expanded input regions, effectively increasing their receptive fields. Figure 2 provides a schematic illustration.

We extend this single-level operation through cascaded decomposition, defined by the following recursive equations:

(6) $$X_{LL}^{\left(i\right)},\;X_{LH}^{\left(i\right)},\;X_{HL}^{\left(i\right)},\;X_{HH}^{\left(i\right)}=\mathrm{WT}\left(X_{LL}^{\left(i-1\right)}\right)$$

(7) $$Y_{LL}^{\left(i\right)},\;Y_{LH}^{\left(i\right)},\;Y_{HL}^{\left(i\right)},\;Y_{HH}^{\left(i\right)}=\mathrm{Conv}\left(W^{\left(i\right)},\;\left(X_{LL}^{\left(i\right)},\;X_{LH}^{\left(i\right)},\;X_{HL}^{\left(i\right)},\;X_{HH}^{\left(i\right)}\right)\right)$$

Here, $X_{LL}^{\left(0\right)}$ serves as the input to the first decomposition layer. The output $X_{H}^{\left(i\right)}$ at each level *i* comprises the three high-frequency sub-bands $\left(X_{LH}^{\left(i\right)},\;X_{HL}^{\left(i\right)},\;X_{HH}^{\left(i\right)}\right)$. Leveraging the linearity of the WT and IWT operations, the multi-level feature aggregation is performed recursively:

(8) $$Z^{\left(i\right)}=\mathrm{IWT}\left(Y_{LL}^{\left(i\right)}+Z^{\left(i+1\right)},\;Y_{LH}^{\left(i\right)},\;Y_{HL}^{\left(i\right)},\;Y_{HH}^{\left(i\right)}\right)$$

where $Z^{\left(i\right)}$ represents the aggregated output from level *i*, and the initial condition for the deepest level *ℓ* is set as $Z^{\left(\ell +1\right)}=0$. Unlike [39], which employs independent normalization for each frequency sub-band, we adopt learnable channel-wise scaling factors after the convolution operations, enabling the network to adaptively balance the contributions from different frequency components.

Figure 2 demonstrates WTCNN application to MobileNetV2’s third inverted residual block, showing two-level decomposition with 3 × 3 kernels.

The WTCNN integration offers two key advantages: (1) Exponential receptive field growth ($2^{\ell }\;\cdot \;k$) with linear parameter increase ($\ell \;\cdot \;4\;\cdot \;c\;\cdot \;k^{2}$) through *ℓ*-level decomposition, and (2) enhanced low-frequency response via recursive WT decomposition of input low frequencies, complementing standard convolution’s high-frequency bias.

These technical merits yield practical benefits including improved scalability versus large-kernel methods, enhanced corruption robustness, and stronger shape (versus texture) sensitivity.

**Figure 3:**
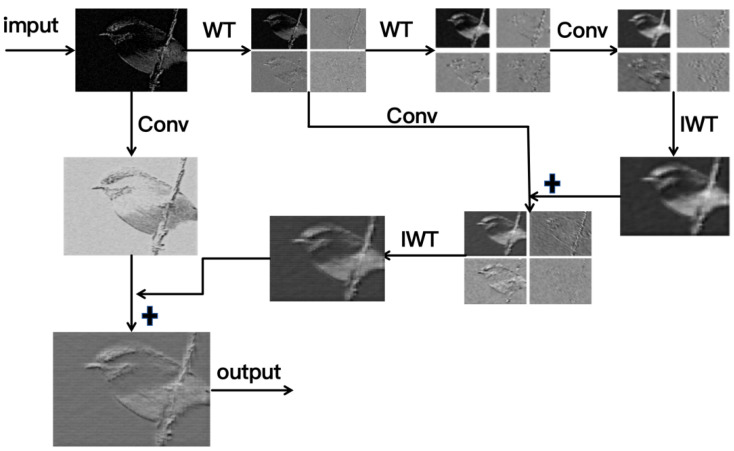
Wavelet transform-based multi-scale feature extraction demonstration.

We further visualize multi-scale feature extraction through wavelet-transformed image features (Figure 3). The decomposition clearly separates image content into distinct frequency bands, producing four characteristic feature maps.

The figure showcases avian image decomposition via 2D discrete wavelet transform. The original image (“WT”) reveals basic silhouette, while subbands highlight: (LL) morphological structures, (LH) vertical feather textures, (HL) horizontal beak/claw edges, and (HH) diagonal microstructures—effectively demonstrating hierarchical feature extraction from macro-shapes to micro-patterns.

### 3.4. Mix Structure Block

**Figure 4:**
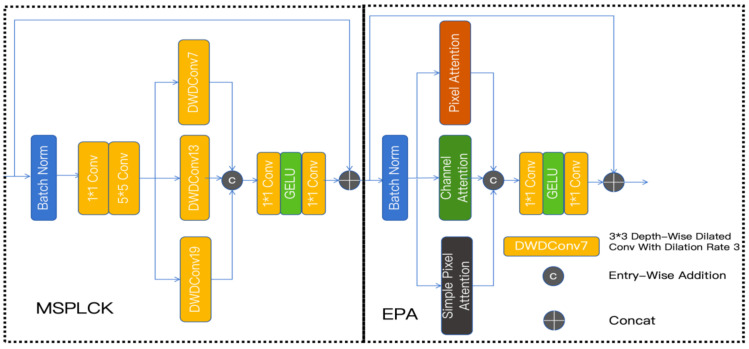
Architecture of the Mix Structure Block, comprising both the multi-scale parallel large convolution kernel module and enhanced parallel attention module.

The mixed structure block integrates two key components: a multi-scale parallel large convolutional kernel module and an enhanced parallel attention module [40], as shown in Figure 4.

#### 3.4.1. Multi-Scale Parallel Large Convolutional Kernel Module (MSPLCK)

The MSPLCK module synergistically combines large receptive fields with multi-scale capabilities. Large-kernel convolutional networks not only provide expanded effective receptive fields but also exhibit significantly stronger shape bias over texture bias. For small object detection tasks, extensive receptive fields are critical—enabling the network to capture the holistic structure of tiny targets through global contextual information, while precisely reconstructing object contours via shape bias.

The processing begins with the original feature map *x*, which undergoes batch normalization:

(9) $$\hat{x}=\mathrm{BatchNorm}\left(x\right)$$

The module’s data flow is mathematically expressed as:

(10) $$\begin{array}{cc} x_{1} & =\mathrm{PWConv}(\hat{x}),\\ x_{2} & =\mathrm{Conv}(x_{1}),\\ x_{3} & =\mathrm{Concat}({\mathrm{DWDConv}}_{19}\left(x_{2}\right),\\ \; & \;{\mathrm{DWDConv}}_{13}\left(x_{2}\right),\\ \; & \;{\mathrm{DWDConv}}_{7}\left(x_{2}\right)) \end{array}$$

where PWConv denotes pointwise convolution, Conv represents standard 5 × 5 convolution, and DWDConv*k* indicates dilated depthwise separable convolution with effective kernel size *k* (7 × 7 kernel with dilation rate 3 for DWDConv19, 5 × 5 kernel with dilation rate 3 for DWDConv13, and 3 × 3 kernel with dilation rate 3 for DWDConv7). The Concat operation merges features along the channel dimension.

This parallel architecture, employing three distinct kernel sizes, facilitates comprehensive multi-scale feature extraction. While larger dilated convolutions provide extensive receptive fields and pronounced shape bias—aiding in locating small targets with long-range dependencies—the smaller variants focus on fine local details and texture information, enhancing discriminative capability for small targets. The concatenation operation triples the feature dimension of $x_{3}$ relative to *x*.

(11) y=x+PWConv(GELU(PWConv(x)))

Subsequently, $x_{3}$ is processed through a multilayer perceptron (MLP) consisting of two pointwise convolutional layers with GELU activation, transforming its feature dimension to match *x*. The MLP output combines with the original input via residual connection, effectively integrating diverse feature types while accommodating the specific representation requirements of small targets.

#### 3.4.2. Enhanced Parallel Attention Module

The Enhanced Parallel Attention (EPA) module represents an innovative architecture that strategically combines complementary attention mechanisms through parallel processing. Our analysis of small target characteristics demonstrates that channel attention mechanisms excel at encoding global semantic information, while pixel attention operations show superior capability in modeling spatially fine-grained features. This architectural design enables simultaneous extraction of both location-specific and globally shared feature representations, making it particularly effective for small target detection tasks.

The EPA framework incorporates three parallel attention pathways operating on batch-normalized feature maps:

(12) $$\hat{x}=\mathrm{BatchNorm}\left(x\right)$$

**Figure 5:**
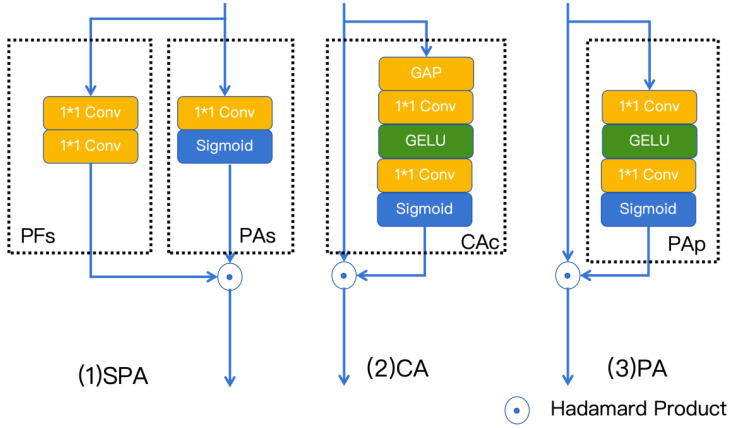
Architectural diagram of the EPA module.

The pixel attention branch specializes in capturing spatially fine-grained features, which are critical for precise localization and classification of small targets. It is shown in Figure 5.

This component comprises two principal submodules: a feature transformation branch ($PF_{s}$) and a spatial attention gate ($PA_{s}$), which operate as follows:

(13) $$\begin{array}{cc} PF_{s} & ={\mathrm{Conv}}_{3\times 3}\left(\mathrm{PWConv}\left(\hat{x}\right)\right) \end{array}$$

(14) $$\begin{array}{cc} PA_{s} & =\sigma (\mathrm{PWConv}\left(\hat{x}\right)) \end{array}$$

(15) $$\begin{array}{cc} F_{s} & =PF_{s}⊙PA_{s} \end{array}$$

where PWConv denotes point-wise convolution operations and σ represents the sigmoid activation function.The extended pixel attention mechanism incorporates additional nonlinear transformations:

(16) $$\begin{array}{cc} PA_{p} & =\sigma (\mathrm{PWConv}\left(\mathrm{GELU}\left(\mathrm{PWConv}\left(\hat{x}\right)\right)\right) \end{array}$$

(17) $$\begin{array}{cc} F_{p} & =\hat{x}⊙PA_{p} \end{array}$$

The channel attention branch provides global contextual modeling through channel-wise feature recalibration:

(18) $$\begin{array}{cc} CA_{c} & =\sigma (\mathrm{PWConv}\left(\mathrm{GELU}\left(\mathrm{PWConv}\left(\mathrm{GAP}\left(\hat{x}\right)\right)\right)\right) \end{array}$$

(19) $$\begin{array}{cc} F_{c} & =\hat{x}⊙CA_{c} \end{array}$$

where GAP denotes global average pooling operations for spatial information aggregation.

Feature integration is achieved through concatenation and nonlinear transformation:

(20) $$\begin{array}{cc} F & =\mathrm{Concat}(F_{s},F_{c},F_{p}) \end{array}$$

(21) $$\begin{array}{cc} y & =x+\mathrm{PWConv}(\mathrm{GELU}(\mathrm{PWConv}(F))) \end{array}$$

The EPA module demonstrates exceptional performance for small object detection due to its dual-capacity architecture. For global semantic information, the channel attention branch generates channel-wise weights to enhance discriminative features of targets, while for spatial details, the pixel attention produces spatial masks to focus on local structures of small objects. This parallel processing of different feature hierarchies through specialized attention mechanisms enables comprehensive modeling of small object characteristics while maintaining computational efficiency.

## 4. Experiment

### 4.1. Datasets

In this paper, we utilize two open source experimental datasets: the DIOR-R dataset [41] and the DOTA dataset [42]. We used the DIOR-R dataset to assess the model’s performance. It comprises 23,190 remote sensing images with 192,512 annotated target instances, covering 20 commonly encountered object categories. All object instances are labeled with an oriented bounding box that indicates their position and orientation in the image. The distribution is 60% for training, 20% for validation, and 20% for testing. The images are resized to 640 × 640 pixels to serve as the model’s input. The DOTA (Dataset for Object Detection in Aerial Images) is a notable large-scale dataset for oriented bounding box (OBB) detection in remote sensing. It encompasses 2806 images, varying in size from 800 × 800 to 4000 × 4000 pixels, with 188,282 instances across 15 categories. This dataset facilitates a comprehensive evaluation of object detection algorithms on diverse aerial images and is employed to verify the framework’s accuracy in oriented object detection tasks.The oriented bounding box (OBB) head predicts the rotation angle θ for each object, parameterized within the range [0, 90°) and represented directly by its value for regression.

### 4.2. Evaluation Metrics

We evaluate model performance using five metrics: Precision (P), Recall (R), mean Average Precision (mAP), F1-score, detection speed (FPS), and mAP for small objects (denoted as mAP(small)). Based on false positives (FP), true positives (TP), false negatives (FN), and true negatives (TN), P and R are defined as follows:

(22) $$\begin{array}{cc} R & =\frac{TP}{TP+FN} \end{array}$$

(23) $$\begin{array}{cc} P & =\frac{TP}{TP+FP} \end{array}$$

IoU threshold is set to 0.5. Subsequently, using the previously obtained recall and precision rates, we plot the Precision–Recall (P-R) curve and compute the Average Precision (AP) as the area under this curve. We evaluate the model using several standard mean Average Precision (mAP) metrics following the COCO evaluation protocol:

- **mAP@0.50**: mAP at a single IoU threshold of 0.50;
- **mAP@0.75**: mAP at a stricter IoU threshold of 0.75;
- **mAP@0.50:0.95**: Average mAP computed over multiple IoU thresholds from 0.50 to 0.95 in steps of 0.05;
- **mAP(small)**: Specifically measures **mAP@0.50** for small objects, defined as those with area less than 32×32 pixels according to the COCO standard.

The final mAP value used for overall model evaluation is defined as the mean value of Average Precision (AP) across all target categories:

(24) $$AP=\int_{0}^{1}P\left(R\right)dR,m\mathrm{AP}=\frac{\sum_{i=1}^{N_{\mathrm{cls}}}AP}{N_{\mathrm{cls}}}$$

where $N_{\mathrm{cls}}$ denotes the number of categories. Furthermore, a higher F1-score indicates stronger robustness of the detection model, defined as follows:

(25) $$F_{1}=2\times \frac{P\times R}{P+R}$$

Additionally, we report inference speed in FPS (Frames Per Second). All metrics are computed on the test set unless otherwise specified.

### 4.3. Experiment Setup

As presented in Table 1, the hardware environment consists of an NVIDIA A100—SXM4—80 GB GPU (NVIDIA, Santa Clara, CA, USA). The software environment runs on Ultralytics 8.3.54 Python—3.8.20 torch—1.9.0 + cu111. We employ the SGD algorithm for end-to-end network optimization, with hyperparameters configured as follows: initial learning rate ($lr_{0}$) of 0.01, final learning rate ($lr_{f}$) of 0.01, batch size of 16, image size (imgsz) of 640, 300 training epochs, 8 workers.

### 4.4. Experiment Results

Table 2, Table 3 and Table 4 present the performance metrics of the YOLOv11-OBB and MS-YOLOv11-OBB models on the DIOR-R and DOTA datasets, respectively. By analyzing the models’ performance across different categories in the DIOR-R dataset, we observe that the improved model achieves significant results in detecting small targets. Specifically, for small target categories such as Storage Tank, and Vehicle, the model’s mAP50 values reach 85.87%, and 81.13%, respectively. These results demonstrate that the model maintains high detection accuracy even when dealing with small and challenging targets. Furthermore, the average mAP50 across all categories reaches 0.8833, further validating the superior overall performance of the model. Additionally, the mAP50 for all categories exceeds 69.61%.

To further compare with the original YOLOv11 and verify the robustness of MS-YOLOv11, we conducted comparative experiments on the DOTA and DIOR-R datasets. The results show that our method achieves better performance, with the improved MS-YOLOv11-OBB model showing overall improvements in Recall, F1-Score, and mAP on the DIOR-R dataset. Compared to the original YOLOv11, MS-YOLOv11 improves the average P, R, F1, and mAP50 by 0.22%, 3.57%, 2.32%, and 2.58%, respectively. Notably, for the Dam category, the original YOLOv11 achieves an mAP of only 56.61%, while MS-YOLOv11 reaches 72.85%, marking a significant improvement of 16.24%. For small target categories such as Storage Tank, Ship, and Vehicle, the model also achieves high precision. Finally, on the DOTA dataset, the proposed MS-YOLOv11 improves P, R, F1, and mAP by 2.75%, 1.98%, 2.19%, and 3.13%, respectively, compared to the original YOLOv11. From these comparisons, we can preliminarily conclude that MS-YOLOv11 performs better in detecting various remote sensing targets and outperforms the original YOLOv11 in terms of precision and accuracy.

**Figure 6:**
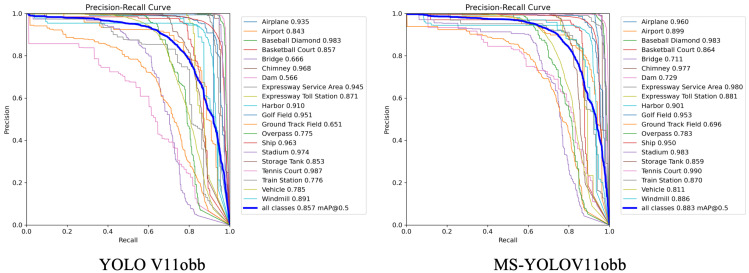
Precision–Recall curve of YOLOV11obb and MS-YOLOV11obb on DIOR-R.

Figure 6 presents the Precision–Recall (P-R) curves for two models, visually reflecting the trade-off between precision and recall at different confidence thresholds. The curve trends indicate that the P-R curve of MS-YOLO V11obb is smoother, closer to the top-right corner of the graph, and the precision decreases more slowly as recall increases. This suggests that MS-YOLO V11bb maintains better performance across different confidence thresholds, demonstrating higher stability and accuracy.

**Figure 7:**
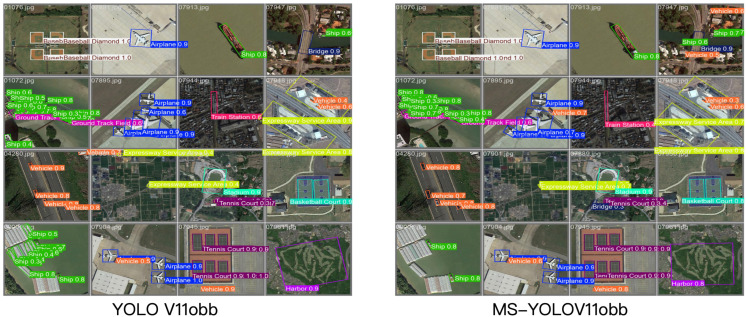
Comparison example of YOLOv11obb and MS-YOLOv11obb test results on DIOR-R.

Lastly, to visually demonstrate the detection performance of MS-YOLOv11, we present some detection results of MS-YOLOv11 and YOLOv11 on the DIOR-R dataset, as shown in Figure 7. YOLOv11 exhibits varying degrees of missed and false detections, while MS-YOLOv11 addresses these issues. Particularly when significant overlap exists between multiple target objects, MS-YOLOv11’s bounding box predictions are more accurate. This further indicates that our improvements enhance the original YOLO performance, contributing to higher accuracy in remote sensing target extraction.

### 4.5. Ablation Experiments

In this work, the Haar wavelet transform is employed for its efficiency and simplicity, although our approach is agnostic to the choice of basis, with others available at the cost of increased computational demand. To further validate the choice of the Haar wavelet and explore the impact of different frequency-domain transformations, we conducted comparative experiments with various wavelet bases under identical experimental settings. As shown in Table 5, while more complex wavelets like Sym8 and Bior2.4 achieved competitive accuracy, possibly due to their higher regularity and better texture representation, the difference in mAP was marginal (<0.5%). Crucially, the Haar wavelet demonstrated a significant advantage in terms of inference speed (FPS), outperforming other bases by a considerable margin. This aligns with our design principle of achieving a optimal balance between accuracy and efficiency for practical applications. Therefore, we conclude that the Haar wavelet is the most suitable choice for the WTConv module in MS-YOLOv11.

According to the ablation study results shown in Table 6, we analyzed the contributions of different components in the MS-YOLOv11 model. By locally enhancing the Backbone with the WTConv module, we improved the mAP from 85.75% to 86.96%, while reducing the number of parameters by approximately 61,591, as the WTConv module replaced the repeatedly used C3K2 modules, achieving a 1.21% increase in accuracy. When independently replacing the neck network with the proposed Mix Structure Block, the mAP increased from 85.75% to 87.81% (an improvement of 2.06%). Overall, while keeping the number of parameters essentially unchanged, MS-YOLOv11 increased the FPS from 302.17 to 447.81, achieving a 2.58% higher mAP compared to the original YOLOv11, and also improved the mAP@75 and mAP@50-95 by 4.33% and 4.24%, respectively. These results demonstrate the effective enhancement of MS-YOLOv11 over the original YOLOv11 performance.

### 4.6. Comparison with Other Detection Models

To validate the superiority of the proposed MS-YOLOv11, we conducted comprehensive comparisons with several representative detection methods on the DIOR-R dataset, including the baseline YOLOv11, YOLOv8, classical oriented object detectors (Rotated Faster R-CNN, Rotated RetinaNet, RoI Transformer, Gliding Vertex, AOPG, GGHL, Oriented RepPoints), and state-of-the-art approaches (LSK-S*, DCFL). As summarized in Table 7, classical methods generally underperformed in remote sensing scenarios, with mAP values ranging from 62.0% to 66.3%. Notably, MS-YOLOv11 achieved the highest mAP of **88.33%**, outperforming YOLOv11 (85.75%) and YOLOv8 (84.96%), and significantly surpassing all other methods. For small object categories—Ship (**SH**), Storage Tank (**STO**), and Vehicle (**VE**)—MS-YOLOv11 attained detection accuracies of **95.03%**, **85.87%**, and **81.13%**, respectively, demonstrating consistent advantages over competing approaches. These results confirm that MS-YOLOv11 not only enhances overall detection performance but also exhibits exceptional capability in small object detection, validating the effectiveness of our architectural improvements.

Benefiting from the WTConv module utilization of 2D Haar Wavelet Transform for multi-level decomposition of input images, it expands the receptive field while extracting both global and detailed features. Secondly, the network further introduces the Mix Structure Block module to enhance small target and multi-scale feature extraction capabilities, acquiring multi-scale features that are ultimately optimized through feature fusion and residual connections. MS-YOLOv11 demonstrates higher adaptability and superior detection performance in remote sensing target detection, effectively addressing issues of missed and false detections. Meanwhile, in terms of speed, MS-YOLOv11 achieves an 145.64 FPS improvement compared to YOLOv11. Furthermore, future research could integrate multi-source remote sensing data and employ techniques like domain adaptive learning to further enhance the accuracy of algorithm and generalization capabilities.

## 5. Conclusions

The proposed MS-YOLOv11 algorithm significantly improves small target detection in remote sensing images by integrating the WTConv module with 2D Haar Wavelet Transform and the Mix Structure Block module. The WTConv module expands the receptive field through multi-level decomposition while jointly extracting global and local features, whereas the Mix Structure Block enhances multi-scale feature fusion. Experimental results demonstrate that MS-YOLOv11 achieved higher detection accuracy on both the DIOR-R and DOTA datasets, with mAP improvements of 2.58% and 3.13%, respectively. The detection performance demonstrates significant improvement in Storage Tank small target category, increasing from 78.50% to 81.13%, a rise of 2.63%. Compared to classical algorithms (e.g., Rotated Faster R-CNN) and the original YOLOv11, the model markedly reduces missed and false detections while delivering more precise bounding box predictions. Future work will focus on incorporating multi-source remote sensing data and domain adaptation learning to further enhance the algorithm’s generalization in complex scenarios.

## Figures and Tables

**Table 1:** Experimental hardware and software environment configuration.

Category	Component	Specification/Version
Hardware Environment	CPU	Intel Xeon Platinum 8369B @ 2.90 GHz (Intel, Santa Clara, CA, USA)
	RAM	512 GB DDR4
	GPU	NVIDIA A100-SXM4-80 GB (x1)
	GPU Memory	80 GB
Software Environment	OS	Ubuntu 20.04.6 LTS
	CUDA	11.1
	cuDNN	8.0.5
	Python	3.8.20
	PyTorch	1.9.0+cu111
	Framework	Ultralytics YOLOv11 (v8.3.54)

**Table 2:** The DOTA dataset results of MS-YOLOV11 OBB(D01: plane, D02: ship, D03: storage tank, D04: baseball diamond, D05: tennis court, D06: basketball court, D07: ground track field, D08: harbor, D09: bridge, D10: large vehicle, D11: small vehicle, D12: helicopter, D13: roundabout, D14: soccer ball field, D15: swimming pool).

Model	Class	Detection Categories															
		**D01**	**D02**	**D03**	**D04**	**D05**	**D06**	**D07**	**D08**	**D09**	**D10**	**D11**	**D12**	**D13**	**D14**	**D15**	**AVG**
YOLOv11obb	P	0.9347	0.9031	0.9337	0.8154	0.9741	0.6699	0.7465	0.8449	0.6605	0.8644	0.6602	0.5024	0.7561	0.6880	0.7112	0.7738
	R	0.8871	0.8572	0.5970	0.7122	0.9001	0.5329	0.5665	0.8359	0.4426	0.8325	0.7171	0.5672	0.5050	0.4929	0.7117	0.6772
	F1	0.9103	0.8795	0.7283	0.7603	0.9231	0.5936	0.6441	0.8403	0.5268	0.8481	0.6820	0.5328	0.6177	0.5561	0.7114	0.7170
	AP	0.9308	0.9016	0.7674	0.7764	0.9462	0.5871	0.6314	0.5333	0.4800	0.8824	0.7221	0.5749	0.5913	0.5021	0.6815	0.7219
MS-YOLOv11obb	P	0.9401	0.7720	0.9421	0.8379	0.9980	0.6610	0.7744	0.8719	0.6875	0.9000	0.7417	0.5218	0.8463	0.6909	0.8933	0.8013
	R	0.8863	0.7531	0.8759	0.7402	0.8651	0.4723	0.5736	0.8107	0.4311	0.8456	0.7749	0.6009	0.8875	0.5432	0.8917	0.6970
	F1	0.9124	0.7624	0.7240	0.7860	0.9001	0.5509	0.6590	0.8402	0.5239	0.8719	0.7580	0.5945	0.6935	0.6082	0.8925	0.7389
	AP	0.9339	0.7885	0.7692	0.8065	0.9254	0.5589	0.6635	0.8686	0.4856	0.9009	0.8019	0.6424	0.6718	0.5518	0.9410	0.7532

Note: P = Precision, R = Recall, AP = Average Precision per class.

**Table 3:** The Dior-R dataset results of MS-YOLOV11 OBB(D01: Airplane, D02: Airport, D03: Baseball Diamond, D04: Basketball Court, D05: Bridge, D06: Chimney, D07: Dam, D08: Expressway Service Area, D09: Expressway Toll Station, D10: Harbor).

Model	Class	Detection Categories (D01–D10)									
		**D01**	**D02**	**D03**	**D04**	**D05**	**D06**	**D07**	**D08**	**D09**	**D10**
YOLOv11obb	P	0.9862	0.8046	0.9764	0.9394	0.8489	0.9767	0.6326	0.9326	0.9708	0.9041
	R	0.9162	0.7786	0.9533	0.7902	0.5552	0.9473	0.5591	0.9167	0.8031	0.8431
	F1	0.9499	0.7914	0.9647	0.8584	0.6713	0.9618	0.5936	0.9246	0.8791	0.8726
	AP	0.9355	0.8429	0.9829	0.8567	0.6657	0.9682	0.5661	0.9449	0.8711	0.9103
MS-YOLOv11obb	P	0.9874	0.8452	0.9835	0.9317	0.8458	0.9729	0.7281	0.9499	0.8900	0.8588
	R	0.9295	0.8358	0.9383	0.8333	0.6434	0.9649	0.6842	0.9356	0.8036	0.8387
	F1	0.9575	0.8405	0.9604	0.8798	0.7309	0.9689	0.7055	0.9427	0.8446	0.8487
	AP	0.9600	0.8993	0.9833	0.8637	0.7113	0.9767	0.7285	0.9803	0.8814	0.9008

**Table 4:** The Dior-R dataset results of MS-YOLOV11 OBB(D11: Golf Field, D12: Ground Track Field, D13: Overpass, D14: Ship, D15: Stadium, D16: Storage Tank, D17: Tennis Court, D18: Train Station, D19: Vehicle, D20: Windmill).

Model	Class	Detection Categories (D11–D20)										
		**D11**	**D12**	**D13**	**D14**	**D15**	**D16**	**D17**	**D18**	**D19**	**D20**	**AVG**
YOLOv11obb	P	0.9316	0.7697	0.9176	0.9391	0.9636	0.9497	0.9828	0.7352	0.9535	0.9315	0.9023
	R	0.8819	0.5579	0.6523	0.9062	0.9474	0.7126	0.9627	0.7921	0.5786	0.8563	0.7955
	F1	0.9060	0.6469	0.7626	0.9224	0.9554	0.8142	0.9727	0.7626	0.7202	0.8923	0.8411
	AP	0.9507	0.6507	0.7750	0.9629	0.9740	0.8527	0.9875	0.7757	0.7850	0.8910	0.8575
MS-YOLOv11obb	P	0.9293	0.8027	0.8514	0.9409	0.9911	0.9339	0.9756	0.8392	0.9173	0.9158	0.9045
	R	0.9113	0.6065	0.6910	0.9098	0.9524	0.7766	0.9634	0.8534	0.6676	0.8839	0.8312
	F1	0.9202	0.6910	0.7629	0.9251	0.9713	0.8480	0.9695	0.8463	0.7728	0.8996	0.8643
	AP	0.9528	0.6961	0.7826	0.9503	0.9833	0.8587	0.9904	0.8702	0.8113	0.8858	0.8833

Note: P = Precision, R = Recall, F1 = F1-Score, AP = Average Precision per class, AVG = Average.

**Table 5:** Performance comparison of MS-YOLOv11 with different wavelet bases on the DIOR-R dataset.

Wavelet Base	mAP@50 (%)	mAP@50:95 (%)	mAP(small) (%)	FPS	Params (M)
Haar (Ours)	88.33	70.90	84.21	447.8	2.87
Db4	88.15	70.75	83.95	421.5	2.87
Db8	88.41	70.88	84.10	415.2	2.87
Sym4	88.28	70.80	83.87	423.1	2.87
Sym8	88.50	71.02	84.35	412.7	2.87
Bior1.3	87.95	70.45	83.70	440.1	2.87
Bior2.4	88.45	70.95	84.28	418.3	2.87

**Table 6:** Ablation study results.

Model Variant	Parameters	mAP@50 (%)	mAP@75 (%)	mAP@50-95 (%)	FPS
YOLOv11-OBB	2,802,647	85.75	71.86	66.66	302.17
YOLOv11-OBB + WTConv	2,741,591	86.96	73.62	68.88	420.35
YOLOv11-OBB + Mix	3,254,025	87.81	75.79	70.50	376.43
MS-YOLOv11	2,865,383	88.33	76.19	70.90	447.81

Note: mAP@50 = mean Average Precision at IoU 50%, FPS = Frames Per Second.

**Table 7:** Comparison with other detection models.

Method	Backbone	SH | STO | VE	mAP
Faster R-CNN-O [43]	R-50	79.4 | 67.6 | 46.2	62.0
SASM [44]	R-50	83.6 | 63.9 | 43.6	62.2
RoI Transformer [45]	R-50	81.2 | 62.5 | 43.8	64.8
Gliding Vertex [46]	R-50	81.0 | 62.5 | 43.2	62.9
KLD [47]	R-50	80.9 | 68.0 | 47.8	64.6
FCOSR [48]	R-50	81.0 | 62.5 | 43.1	63.4
Oriented RepPoints [49]	R-50	85.1| 65.3 | 48.0	66.3
LSKNet-S * [50]	LSK-S	81.2 | 70.9 | 49.8	71.6
† DCFL [51]	R-50	- | - | 50.9	71.0
YOLO V8obb * [52]	CSP	- | - | -	86.3
YOLO V8obb	CSP	94.91 | 81.95 | 79.78	84.96
YOLO V11obb	CSP	96.29 | 85.27 | 78.50	85.75
MS-YOLOv11obb	CSP	95.03 | 85.87 | 81.13	88.33

Note: SH = Ship, STO = Storage Tank, VE = Vehicle, mAP = mean Average Precision. * denotes improved versions of the baseline models. † indicates the model presented in the cited reference.

## Data Availability

Data is contained within the article.
